# Endoscopic endonasal transtuberculum-transplanum-translamina terminalis approach for third ventricle craniopharyngioma: 2D operative video

**DOI:** 10.3171/2026.7.FOCVID2617

**Published:** 2026-10-01

**Authors:** Simona Serioli, Iacopo Dallan, Michele Caniglia, Daniel M. Prevedello, Matteo Barucco, Lorena di Girolami, Francesco Acerbi

**Affiliations:** 1 Skull Base and Rhino-Orbital Surgery Unit, Azienda Ospedaliero-Universitaria Pisana, Pisa;; 2 Department of Neuroscience and Surgery of the Nervous System, Papa Giovanni XXIII Hospital, Bergamo;; 3 Technology for Health PhD Program, University of Brescia;; 4 Division of Neurosurgery, Pisa University Hospital, Pisa, Italy; and; 5 Department of Neurosurgery, The Ohio State University, Wexner Medical Center, Columbus, Ohio

**Keywords:** extended endonasal endoscopic approach, papillary craniopharyngioma, third ventricle, translamina terminalis

## Abstract

The management of craniopharyngiomas remains a challenge for multidisciplinary pituitary teams. Their infiltrative behavior, particularly in the hypothalamic region, renders arachnoid and extracapsular dissection technically demanding and may result in irreversible impairment of patients’ quality of life. The extended endoscopic endonasal approach has gained widespread acceptance, especially for sellar and suprasellar lesions due to superior magnification and spatial resolution, demonstrating an inverse relationship between resection extent and postoperative morbidity. This video illustrates the endoscopic resection of a third ventricle craniopharyngioma through the suprachiasmatic corridor using a transtuberculum-transplanum-translamina terminalis approach, emphasizing the pertinent surgical anatomy.

The video can be found here: https://stream.cadmore.media/r10.3171/2026.7.FOCVID2617

## Transcript

### 0:22 Patient Clinical Presentation and Imaging.

We present the case of a 58-year-old man with no remarkable clinical history. In the last 6 months, he reported progressive visual acuity and visual field deficit, accompanied by weight gain. Preoperative visual testing demonstrated bitemporal hemianopia, while endocrine evaluation revealed normal pituitary function. Brain gadolinium-enhanced MRI documented a pure heterogeneous intraventricular mass localized entirely within the third ventricle.

### 0:54 Rationale for the Procedure.

Surgical resection was recommended due to the progression of symptoms, the risk of obstructive hydrocephalus, and the need for diagnosis. Given the tumor extension, an endoscopic endonasal approach was considered more advantageous than a transcranial route.

An endoscopic endonasal transtuberculum-transplanum-translamina terminalis approach^1^ was chosen.

It is characterized by an anterosuperior trajectory between the vascular complex and optic chiasm. The floor of the third ventricle and the infundibular area could be partially hidden from this perspective, and this kind of manipulation requires careful maneuvering. However, the direct visualization assured by an anteroposterior route may reduce the need for lateral traction of the hypothalamic wall.

### 1:43 Anatomical Landmark.

After the transethmoidal sphenoidotomy, craniectomy was extended from the sellar prominence to the planum sphenoidale, considering the supraoptic recess and medial optic-carotid recess as lateral limits.^2^

After a T-shaped incision of the periosteum preserving the superior intercavernous sinus, the optic chiasm represents the key landmark for the definition of the supra- and infrachiasmatic corridors.

The arachnoid was opened sharply, and careful dissection between the anterior cerebral artery complex and the superior surface of the optic chiasm is mandatory to reach the lamina terminalis, which represents the anterior wall of the third ventricle.

The endoscopic endonasal approach provides panoramic, close-up visualization, if necessary with the aid of angled optics, facilitating inspection of the anterior third ventricle.

The risks of the procedure were CSF leak, vascular injury, diabetes insipidus, worsening of visual impairment due to direct optic nerve/chiasm manipulation, thermal/microvascular injury, and ischemia, hypothalamic dysfunction, meningitis, abulia, and apathy.

### 2:56 Alternative Strategies.

Alternative strategies include biopsy or function-preserving surgery, particularly when the tumor is invasive and closely related to the hypothalamus or optic apparatus, followed by BRAF/MEK-targeted therapy,^3^ if a BRAF V600E–positive papillary craniopharyngioma (found in approximately 90%–95% of papillary craniopharyngiomas) is confirmed. This case was treated 6 years ago, when current available data were not yet well established.

Other surgical options involve a transcranial approach (transfrontal/subfrontal translamina terminalis and interhemispheric transcallosal approach).

Regarding the other surgical options,^4,5^ the transcallosal approach is a unilateral craniocaudal corridor based on tumor dimension, caliber of foramen, and associated hydrocephalus. There is a high risk of bridging vein injury during the dissection, and memory and cognitive issues due to fornix manipulation. The deep corridor is characterized by limited maneuverability, where visualization is inherently limited to a straight line of sight through the operating microscope. Moreover, experience in this kind of microsurgical approach is required.

Subfrontal/bifrontal approach is indicated for small anteroinferior lesions, with craniocaudal midline trajectory to the third ventricle. Depending on the patient’s anatomy, it may require frontal sinus cranialization with associated reconstruction. There is a high risk of damage perforating arteries and brain retraction injury.

The pterional/orbitozygomatic approach offers an antero- and craniocaudal trajectory that implicates excessive manipulation of neurovascular structures and limited visualization of the ipsilateral portion of the third ventricle.

### 4:48 Surgical Setup.

The patient was positioned supine and tilted to the right side, with the head slightly rotated to the right and secured in a Mayfield three-pin head holder. Sterile abdominal and lateral thigh fields were prepared for harvesting autologous fat and fascia lata grafts to be used as part of the multilayer skull base reconstruction. Electromagnetic neuronavigation guidance, visual evoked potential monitoring, and an ultrasonic aspirator were employed throughout the procedure.

### 5:19 Key Surgical Steps.

A binostril endoscopic approach was performed using a 0° endoscope, with lateralization of the middle and superior turbinates and wide sphenoidotomy. A nasoseptal flap was harvested for the reconstruction phase. After the removal of the anterior wall of the sphenoid sinus, the sphenoid cavity was flattened using a high-speed drill with a diamond burr to better visualize the bony prominences. After obtaining an eggshell consistency, thin bone was carefully removed.

After coagulation of the anterior intercavernous sinus,^2^ a transverse incision of the periosteum was made over the planum sphenoidale, followed by a midline cut at the level of the tuberculum. Using a dissector, the chiasm cistern arachnoid was opened sharply, offering identification of the optic chiasm, pituitary stalk, and superior hypophyseal arteries.

To better define the extension of the suprachiasmatic corridor, the anterior cerebral complex was dissected from the superior surface of the optic chiasm, in order to access the lamina terminalis cistern. The lesion was located immediately posterior to the lamina terminalis, with the optic chiasm displaced inferiorly, and an intact pituitary stalk.

Carefully pushing the gyri rectus and the anterior cerebral complex, the lamina terminalis was exposed and opened.

A cleavage plane with the wall of the third ventricle was defined using a dissector and aspirator.

The tumor, characterized by a soft consistency, was removed using a bimanual technique.

An ultrasonic aspirator was needed to remove parts of the tumor having a more solid consistency. Progressive debulking along the long axis of the lesion was achieved by alternating the use of curettes. A small remnant was left to avoid excessive downward displacement of the optic chiasm, while still allowing for a relative blind dissection.

Using a 45° endoscope, a final view showed satisfactory resection of the lesion, identifying the foramen of Monro and the choroid plexuses. Visual evoked potentials did not show any alterations or significant change throughout the surgical procedure.

After meticulous hemostasis, the skull base was reconstructed using a dural substitute as an inlay graft, and autologous fat fixed with fibrin glue. Fascia lata was used as an onlay graft covered by a nasal flap.

### 8:47 Postoperative Imaging and Clinical Outcome.

Postoperative MRI documented the presence of a small residue left intentionally due to its strong adhesion^6^ to the third-floor ventricle. No postoperative prophylactic spinal drainage was used. Nasal swabs were removed on the 2nd postoperative day under endoscopic guidance, with no signs of cerebrospinal fluid fistulas. Histopathological examination revealed a WHO grade I papillary craniopharyngioma, characterized by a BRAF-V600E mutation. During clinical follow-up, the patient documented improvement of the bitemporal hemianopia with no hypothalamic obesity or sleep-wake disturbances. Four years after surgery, the patient is clinically well with stable residual tumor.

## Disclosures

D. Prevedello reported consulting for Leica, Stryker, Integra, and BK Medical; and royalties from KLS Martin, ACE Medical, and Mizuho.

## Author Contributions

Primary surgeon: Caniglia. Assistant surgeon: Dallan, Barucco. Editing and drafting the video and abstract: Serioli, Dallan, di Girolami, Acerbi. Critically revising the work: Serioli, Dallan, Prevedello, Acerbi. Reviewed submitted version of the work: Serioli, Dallan, Prevedello. Approved the final version of the work on behalf of all authors: Serioli. Supervision: Dallan, Prevedello, Acerbi.

## Supplemental Information

### Patient Informed Consent

The necessary patient informed consent was obtained in this study.
